# Using a Convolutional Neural Network and Convolutional Long Short-term Memory to Automatically Detect Aneurysms on 2D Digital Subtraction Angiography Images: Framework Development and Validation

**DOI:** 10.2196/28880

**Published:** 2022-03-16

**Authors:** JunHua Liao, LunXin Liu, HaiHan Duan, YunZhi Huang, LiangXue Zhou, LiangYin Chen, ChaoHua Wang

**Affiliations:** 1 Department of Neurosurgery West China Hospital Sichuan University Chengdu China; 2 College of Computer Science Sichuan University Chengdu China; 3 School of Science and Engineering The Chinese University of Hong Kong Shenzhen China; 4 School of Automation Nanjing University of Information Science and Technology Nanjing China

**Keywords:** convolutional neural network, convolutional long short-term memory, cerebral aneurysm, deep learning

## Abstract

**Background:**

It is hard to distinguish cerebral aneurysms from overlapping vessels in 2D digital subtraction angiography (DSA) images due to these images’ lack of spatial information.

**Objective:**

The aims of this study were to (1) construct a deep learning diagnostic system to improve the ability to detect posterior communicating artery aneurysms on 2D DSA images and (2) validate the efficiency of the deep learning diagnostic system in 2D DSA aneurysm detection.

**Methods:**

We proposed a 2-stage detection system. First, we established the region localization stage to automatically locate specific detection regions of raw 2D DSA sequences. Second, in the intracranial aneurysm detection stage, we constructed a bi-input+RetinaNet+convolutional long short-term memory (C-LSTM) framework to compare its performance for aneurysm detection with that of 3 existing frameworks. Each of the frameworks had a 5-fold cross-validation scheme. The receiver operating characteristic curve, the area under the curve (AUC) value, mean average precision, sensitivity, specificity, and accuracy were used to assess the abilities of different frameworks.

**Results:**

A total of 255 patients with posterior communicating artery aneurysms and 20 patients without aneurysms were included in this study. The best AUC values of the RetinaNet, RetinaNet+C-LSTM, bi-input+RetinaNet, and bi-input+RetinaNet+C-LSTM frameworks were 0.95, 0.96, 0.92, and 0.97, respectively. The mean sensitivities of the RetinaNet, RetinaNet+C-LSTM, bi-input+RetinaNet, and bi-input+RetinaNet+C-LSTM frameworks and human experts were 89% (range 67.02%-98.43%), 88% (range 65.76%-98.06%), 87% (range 64.53%-97.66%), 89% (range 67.02%-98.43%), and 90% (range 68.30%-98.77%), respectively. The mean specificities of the RetinaNet, RetinaNet+C-LSTM, bi-input+RetinaNet, and bi-input+RetinaNet+C-LSTM frameworks and human experts were 80% (range 56.34%-94.27%), 89% (range 67.02%-98.43%), 86% (range 63.31%-97.24%), 93% (range 72.30%-99.56%), and 90% (range 68.30%-98.77%), respectively. The mean accuracies of the RetinaNet, RetinaNet+C-LSTM, bi-input+RetinaNet, and bi-input+RetinaNet+C-LSTM frameworks and human experts were 84.50% (range 69.57%-93.97%), 88.50% (range 74.44%-96.39%), 86.50% (range 71.97%-95.22%), 91% (range 77.63%-97.72%), and 90% (range 76.34%-97.21%), respectively.

**Conclusions:**

According to our results, more spatial and temporal information can help improve the performance of the frameworks. Therefore, the bi-input+RetinaNet+C-LSTM framework had the best performance when compared to that of the other frameworks. Our study demonstrates that our system can assist physicians in detecting intracranial aneurysms on 2D DSA images.

## Introduction

The prevalence of cerebral aneurysms in the general population is approximately 2% to 3% [1]. When an intracranial aneurysm ruptures, it may bleed into the brain parenchyma, causing a hemorrhage of the cerebral parenchyma, or, more commonly, it bleeds into the subarachnoid space and causes a subarachnoid hemorrhage (SAH). An SAH is a catastrophic event with a mortality rate of 25% to 50%. Nearly 50% of SAH survivors have permanent disabilities; only approximately one-third of patients with SAH have good prognoses [2,3]. Hence, it is crucial to detect and treat aneurysms as early as possible. The gold standard for diagnosing cerebral aneurysms is digital subtraction angiography (DSA). The application of 3D DSA has dramatically improved the diagnostic accuracy for aneurysms. However, as many hospitals lack the technical and reconstitution equipment for 3D DSA, especially in low-income countries, radiologists in these hospitals have to diagnose cerebral aneurysms by using 2D DSA images. Unlike 3D images, 2D DSA images lack spatial information, and it is difficult for radiologists to distinguish aneurysms from overlapping vessels in 2D DSA images. Therefore, the assessment of these 2D DSA images is usually subjective and may be influenced by the experience of radiologists.

In recent years, image recognition via deep learning for diagnostic imaging has achieved good performance in various medical fields, such as skin cancer, retinopathy, pneumonia, and gastric cancer [4]. Deep learning represents a new machine learning method that enables a machine to analyze various training images, so that it can extract specific clinical features [5]. Based on the cumulative clinical features, a machine can prediagnose newly acquired clinical images.

A convolutional neural network (CNN) is a type of deep learning model for processing data that have a grid pattern, such as images. CNNs were inspired by the organization of the animal visual cortex [6,7] and designed to automatically and adaptively learn spatial hierarchies of characteristics from low- to high-level pictures. CNNs have achieved good performance in several medical fields, such as lesion detection [8] and classification [9].

Convolutional long short-term memory (C-LSTM) networks [10] have advantages over feedforward neural networks, as they can discover the hidden structures of medical time signals. C-LSTM networks can perform pattern recognition analyses on medical time series data and have obtained high accuracies in the classification of medical signals [11,12].

Recent studies have used deep learning methods for detecting cerebral aneurysms in 2D DSA images, but these works have some limitations. Podgoršak et al [13] modified the Visual Geometry Group network—a network used for classification—into a network suitable for semantic segmentation tasks for detecting aneurysms. The data set of their study was composed of positive case data for aneurysms, and its false-positive rate has not been evaluated. Jin et al [14] used a bidirectional C-LSTM network to segment aneurysms; although the network’s patient-level sensitivity was 97.7%, the average number of false positives per sequence was as high as 3.77. Liao et al [15] used a C-LSTM network to extract time information when detecting aneurysms but did not consider the relationships among DSA images from different aspects of the same patient. Duan et al [16] combined frontal and lateral DSA images for detection but did not use the timing information of the DSA sequence. This method requires an additional false-positive correction algorithm for correcting the results. Therefore, the existing deep learning–based aneurysm detection methods still need to be improved.

To solve the aforementioned problems, we combined a CNN for acquiring spatial information and a C-LSTM network for learning temporal information to detect aneurysms in 2D DSA images.

Posterior communicating artery (PCoA) aneurysms are one of the most common aneurysms encountered by neurosurgeons and neurointerventional radiologists and are the second most common aneurysms overall (25% of all aneurysms), representing 50% of all internal carotid artery (ICA) aneurysms [17]. Hence, to solve the problem of data deficiency, we focused on PCoA aneurysms to (1) construct a deep learning diagnostic system to improve the ability to detect PCoA aneurysms on 2D DSA images and (2) validate the efficiency of the deep learning diagnostic system in 2D DSA aneurysm detection.

**Figure 1:**
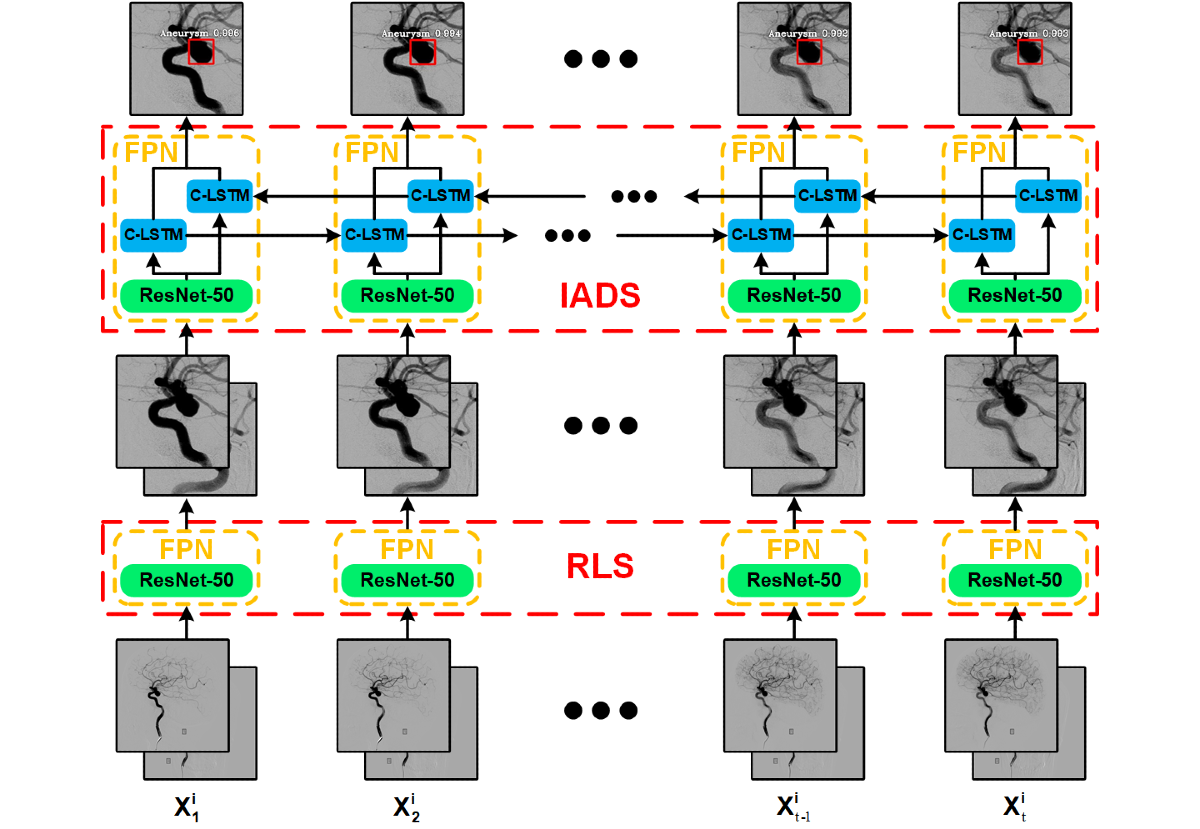
The flowchart of the deep learning diagnostic system. “X^i^_t_” represents the tth frame in the digital subtraction angiography sequence of the ith patient. C-LSTM: convolutional long short-term memory; FPN: feature pyramid network; IADS: intracranial aneurysm detection stage; ResNet: residual deep neural network; RLS: region localization stage.

This deep learning diagnostic system includes a region localization stage (RLS) and an intracranial aneurysm detection stage (IADS). The RLS is used to automatically locate a specific detection area, and in the IADS, the system conducts aneurysm detection for the area images outputted in the RLS. The cascading framework flowchart is shown in Figure 1.

The main contributions of this paper can be summarized as follows. First, the bi-input network framework of the IADS increases the amount of information and then combines spatial-temporal information through feature pyramid networks (FPNs) [18], with a residual deep neural network (ResNet) [19] and bidirectional C-LSTM network acting as the backbone. This greatly improves the accuracy and efficiency of aneurysm detection. Second, our proposed method can achieve low false-positive rates without the need for a false-positive correction algorithm.

## Methods

### Ethics Approval

This retrospective study was approved (number 20220310005) by the institutional review board of West China Hospital, Sichuan University, Chengdu, China, with a waiver of written informed consent.

### Study Design

A total of 586 patients who underwent DSA examination and had identified PCoA aneurysms from January 2014 to December 2019 in West China Hospital were included in this study. All of the PCoA aneurysms were double confirmed via 3D DSA. The main inclusion criterion stipulated that patients were diagnosed with PCoA aneurysms via DSA. The exclusion criteria consisted of the following: (1) patients lacking lateral frontal DSA images; (2) patients with arteriovenous malformations, arteriovenous fistulas, or moyamoya disease; (3) patients with treated aneurysms; and (4) patients with aneurysms in other locations.

The obtained images were in DICOM format, which requires a large memory space. To decrease the computational load and improve usability, we converted the images to PNG format in model training and testing.

**Figure 2:**
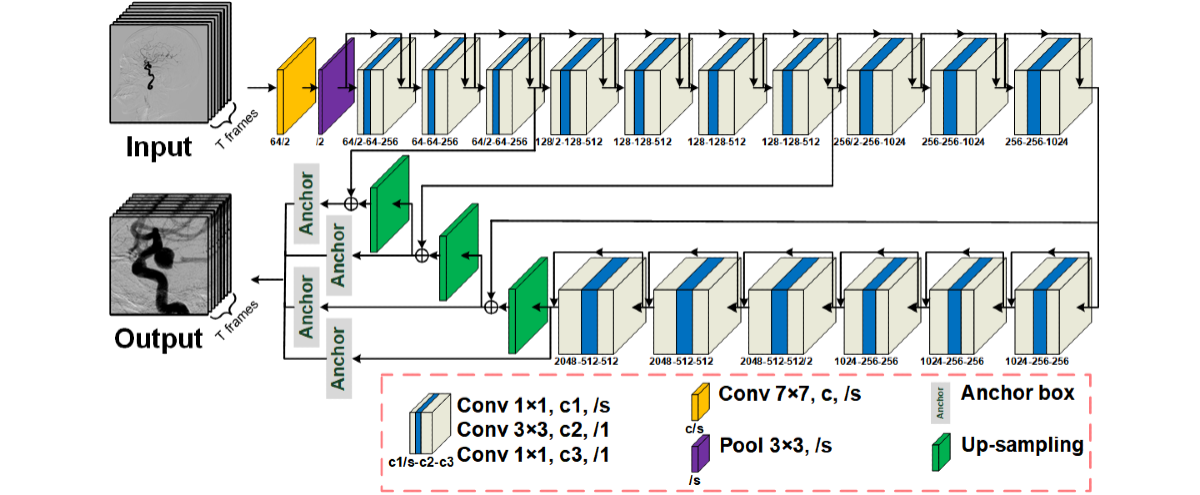
The network architecture of the RLS. “Conv f×f, c, /s” represents a 2D convolutional layer with a kernel size of f×f, a c number of channels, and an s number of strides, which is defaulted to 1. “Pool f×f, /s” denotes the maximum pooling layer, which has a filter size of f and an s number of strides. The “anchor” is used to predict the PCoA region, and “up-sampling” refers to nearest neighbor up-sampling with an up-sampling rate of 2. PCoA: posterior communicating artery; RLS: region localization stage.

Two experienced radiologists identified 6 to 12 frameworks for 2D DSA images, which provided sufficient visualization of the PCoA region. Manual annotations were performed for the identification of aneurysms, vessel overlaps, and PCoA regions. To augment the training data, each image was rotated randomly between 0° and 359°. The data set was divided into the following three parts: the training set, validation set, and test set. The training set was used to train the algorithm, the validation set was used for model selection, and the test set was used for the assessment of the final chosen model. To obtain a reliable and stable model, this study adopted 5-fold cross-validation, during which the data set was divided into 5 parts; 4 parts were used for training and 1 part was used for validation. The mean value of the 5 results was used as the algorithm accuracy. The advantage of cross-validation is that it can make full use of limited data to find suitable model parameters and prevent overfitting. Raw 2D DSA images usually have large resolutions. Initially, the detection of intracranial aneurysms was based on original 2D DSA images, and the large resolution of the original 2D DSA images resulted in extra time consumption and interference. Specifically, researchers have attempted to avoid large resolution–related problems by manually locating detection areas requiring considerable amounts of work. In our case, we used the RLS to automatically locate specific detection regions of raw 2D DSA sequences, as shown in Figure 2. This method can be used to reduce the interference in aneurysm detection. In theory, region localization can be performed to locate any ICA region, but we could only prove the feasibility of using the RLS to identify PCoA regions due to the limitations of the data set. As shown in Figure 2, this architecture uses a raw 2D DSA sequence as input. The ResNet-50–based [19] FPN sends the features extracted from each frame to anchor boxes [20] to predict the PCoA region. The detector outputs 6n parameters in which “6” represents the bounding box’s x-coordinate, y-coordinate, width, height, classification label, and confidence for classification and “n” refers to the n objects detected in the RLS. The bounding box with the highest prediction confidence was applied to other frameworks in the DSA sequence, and it outputted the PCoA region sequence. Moreover, to connect with the IADS, each frame of the output sequence was resized to 288×288 pixels during the RLS.

ResNet was the winner of the 2015 ImageNet Large Scale Visual Recognition Challenge for image classification [19]. It has several advantages over traditional CNNs, as follows: (1) it accelerates the training speed of deep networks; (2) instead of widening the network, it increases the depth of the network, resulting in fewer extra parameters; (3) the residual block inside ResNet uses jump connections to alleviate the problem of gradient disappearance resulting from the increase in the depth of the deep neural network; and (4) it achieves higher accuracy in network performance, especially in image classification [19]. Due to the excellent performance of ResNet, it has been widely used in various medical imaging tasks [21-23].

C-LSTM is a variant of long short-term memory (LSTM) that has a convolution operation inside of the LSTM cell. Both models are special kinds of recurrent neural networks that are capable of learning long-term dependencies. The main difference between C-LSTM and LSTM is the number of input dimensions. Using LSTM to process image sequences with temporal information requires converting 3D data to 2D data, which inevitably results in the loss of information. C-LSTM networks inherit the advantages of traditional LSTM networks and are very suitable for the analysis of spatiotemporal data due to their internal convolution structure. Therefore, many studies use C-LSTM to process medical image sequences [11,12,24].

Detecting objects at different scales, particularly small objects, is challenging. FPNs combine low-resolution, semantically strong features with high-resolution, semantically weak features via a top-down pathway and lateral connections. FPNs have rich semantics at all levels and are built quickly from a single-input image scale without sacrificing representational power, speed, or memory [18].

Object detection algorithms have 2 classic structures—1-stage and 2-stage algorithms. Compared to the 1-stage algorithm, the 2-stage algorithm has 1 more step for solving the problem of class imbalance. Therefore, the 2-stage algorithm is more time-consuming. Lin et al [25] constructed RetinaNet by combining ResNet, FPNs, and fully convolutional networks [26]. The RetinaNet algorithm solves the problem of class imbalance by using the focal loss function instead of the proposal extraction step, thereby greatly improving the detection speed with high accuracy. Because of the excellent performance of RetinaNet, it is widely used in object detection tasks involving medical images [27-29].

We compared the following three structures in the IADS: (1) RetinaNet [25], which uses single-frame images as input; (2) RetinaNet+C-LSTM [15], which is based on RetinaNet and uses C-LSTM to extract bidirectional time information and take frontal or lateral DSA sequences as input; and (3) bi-input+RetinaNet [16], which combines frontal and lateral DSA sequences together as input.

**Figure 3:**
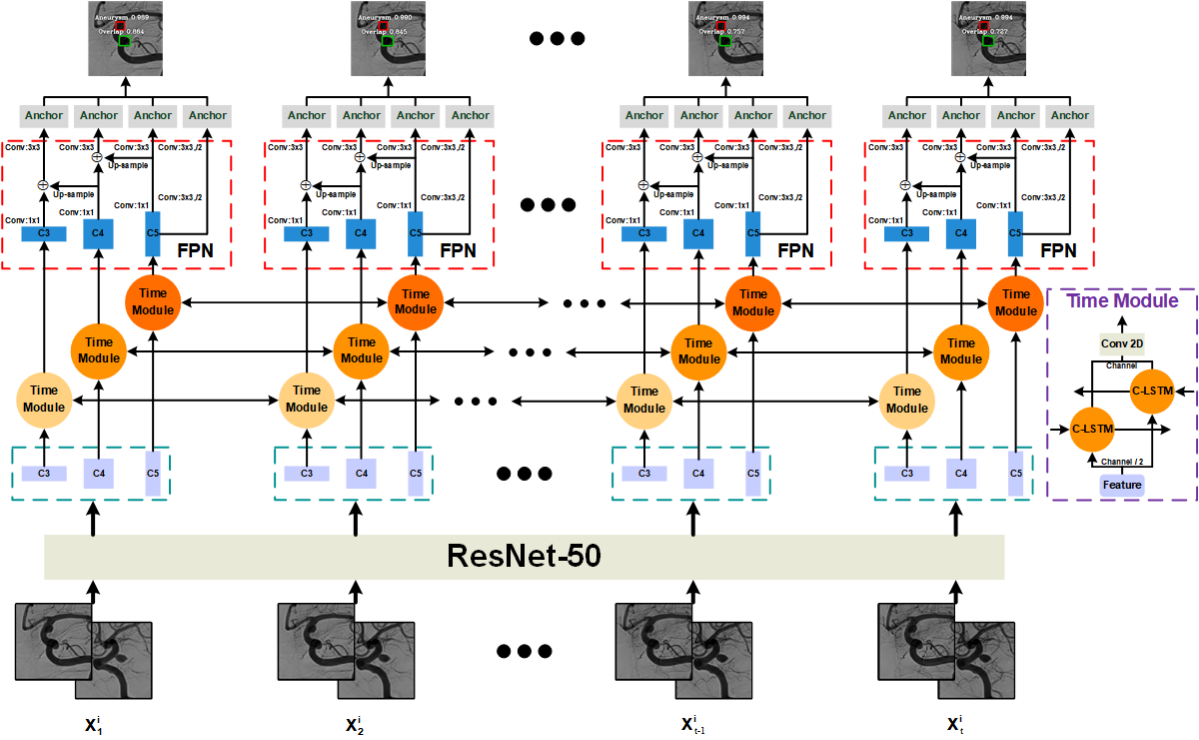
The network architecture of the IADS. “X^i^_t_” represents the tth frame in the DSA sequence of the ith patient. “Conv: f×f, /s” represents a convolutional layer with a kernel size of f×f and an s number of strides, where s is defaulted to 1. The channel of the convolutional layer defaults to 256. “C3,” “C4,” and “C5” represent the 3-layer features of ResNet-50. “Up-sample” refers to nearest neighbor up-sampling with an up-sampling rate of 2. The “anchor” denotes the anchor box, which uses the features to output the detection result. C-LSTM: convolutional long short-term memory; Conv 2D: 2D convolution; DSA: digital subtraction angiography; FPN: feature pyramid network; IADS: intracranial aneurysm detection stage; ResNet: residual deep neural network.

As shown in Figure 3, the target sequence of the PCoA region and its corresponding frontal or lateral sequence were concatenated as a 6-channel image sequence in which the target sequence occupies the first 3 channels. ResNet-50 extracted individual spatial features from each 6-channel frame in the input sequence. In total, 3 feature layers were selected for temporal feature extraction by using bidirectional C-LSTM, namely C3, C4, and C5, which had 512, 1024, and 2048 channels, respectively. It should be noted that the number of channels in the C-LSTM network was set as half of the input. After extracting the temporal information, we concatenated the features of the forward C-LSTM network and the reverse C-LSTM network and sent them to the FPN for further extraction. Anchor boxes identified intracranial aneurysms and overlapping blood vessels based on the features extracted by the FPN. To make the detection results more reliable, the detector only outputted the predicted objects with a confidence level of >0.6.

All models were trained and tested with a Keras [30] deep learning framework on an NVIDIA GTX 1080Ti graphics processing unit (11GB GDDR5X; NVIDIA Corporation). We used the data in the training set to train the region localization and intracranial aneurysm detection algorithms, and the initial learning rate of each step in the training process was set to 3×10^−6^ for the RLS and 1×10^−4^ for the IADS. The Adam optimization method [31] was adopted, and the learning rate was dynamically adjusted with the training progress. If the variation in the range of loss in 2 consecutive epochs was less than 1×10^−4^, then the learning rate was reduced by a factor of 10. This method achieved the local optimum of the training process.

The loss function for object classification used focal loss [25]. This loss function reduced the weight of the large number of simple negative samples in training, thereby solving the problem of a serious imbalance in the ratio of positive to negative samples in object detection tasks. The focal loss was defined as follows:



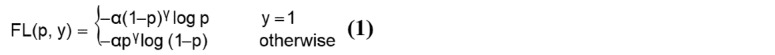



where “FL” denotes focal loss, “α” denotes the balanced parameter used to balance the proportional inequality of positive and negative samples, “γ” denotes the downweighted rate, “p” represents prediction confidence, and “yϵ{±1}” is the ground truth class. When γ was >0, the loss function reduced the loss of easy-to-classify samples and thus focused more on difficult and misclassified samples. Specifically, we used an α of .25 and a γ of 2.0 in the training process.

Smooth L1 loss [25] was used as the loss function for bounding box regression. As a commonly used loss function in regression tasks, smooth L1 loss can limit the gradient value from the following two aspects to prevent training failure: (1) when the difference between the predicted value and the ground truth was too large, the gradient value was not too large, and (2) when the predicted value was very close to the ground truth, the gradient value was small enough. This loss function was defined as follows:



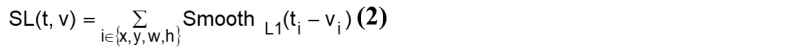



in which



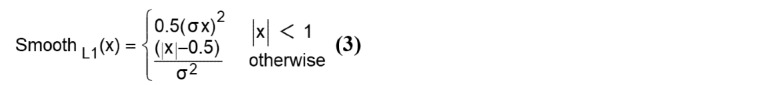



where “SL” denotes smooth L1 loss, “t” denotes the bounding box of the predicted object, “v” represents the bounding box of the ground truth, and “σ” is the weighted factor. A σ of 3.0 was used in the training process.

### Statistical Analysis

Statistical analyses were performed by using statistical software (SPSS version 22.0; IBM Corporation). We used the 5-fold cross-validation strategy with mean average precision (mAP) values to assess the accuracy of intracranial aneurysm and overlap classification. The bounding box regression task was evaluated based on the smooth L1 loss. A confusion matrix, receiver operating characteristic (ROC) curves, and area under the curve (AUC) values were used to assess the abilities of different frameworks. For ROC curves, comparisons of AUC values (with SEs and 95% CIs) were made by using a nonparametric approach [32]. A total of 20 patients with PCoA aneurysms (test set) and 20 patients without aneurysms were used to evaluate the performance of each framework and the human experts, who had 20 years of experience. True positives, true negatives, false positives, and false negatives were used to calculate sensitivity, specificity, and accuracy, which were determined based on the optimal threshold from the Youden index. The adjusted Wald method was used to determine the 95% CIs of the accuracy, sensitivity, and specificity values from the contingency tables [33].

## Results

During the RLS, the system only needs to perform the simple task of determining the valid coarse regions. The accuracy of region localization for the test set was 100%, which proves that this method accurately located the PCoA regions from the original DSA images.

**Figure 4:**
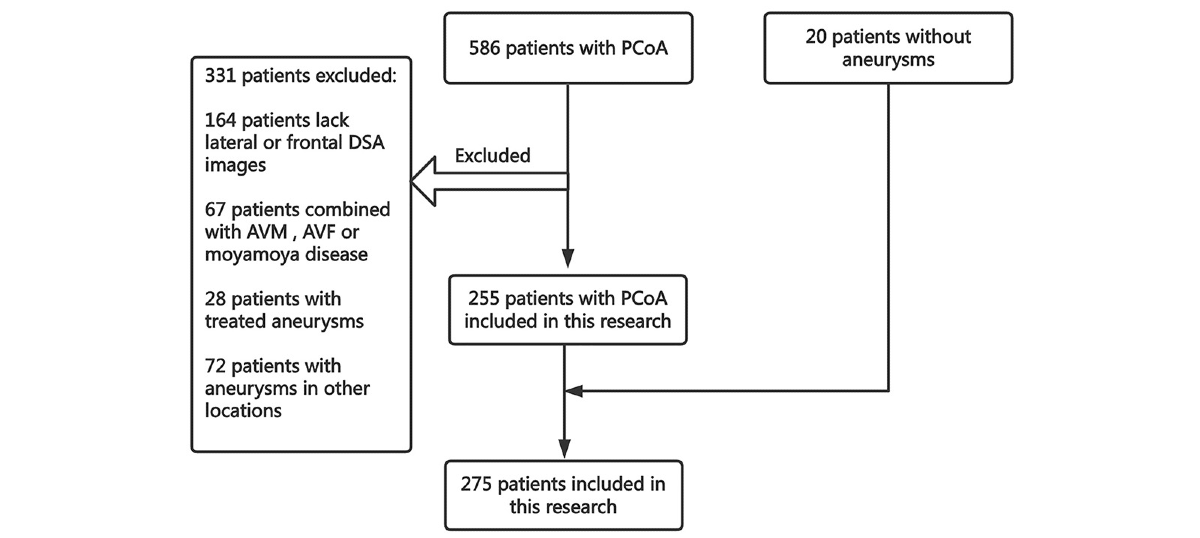
Flowchart of enrollment information for included patients. AVF: arteriovenous fistula; AVM: arteriovenous malformation; DSA: digital subtraction angiography; PCoA: posterior communicating artery.

Of the 275 patients included in this study, 255 had PCoA aneurysms, and 20 did not have aneurysms. A flowchart of the enrolled patients is shown in Figure 4.

**Figure 5:**
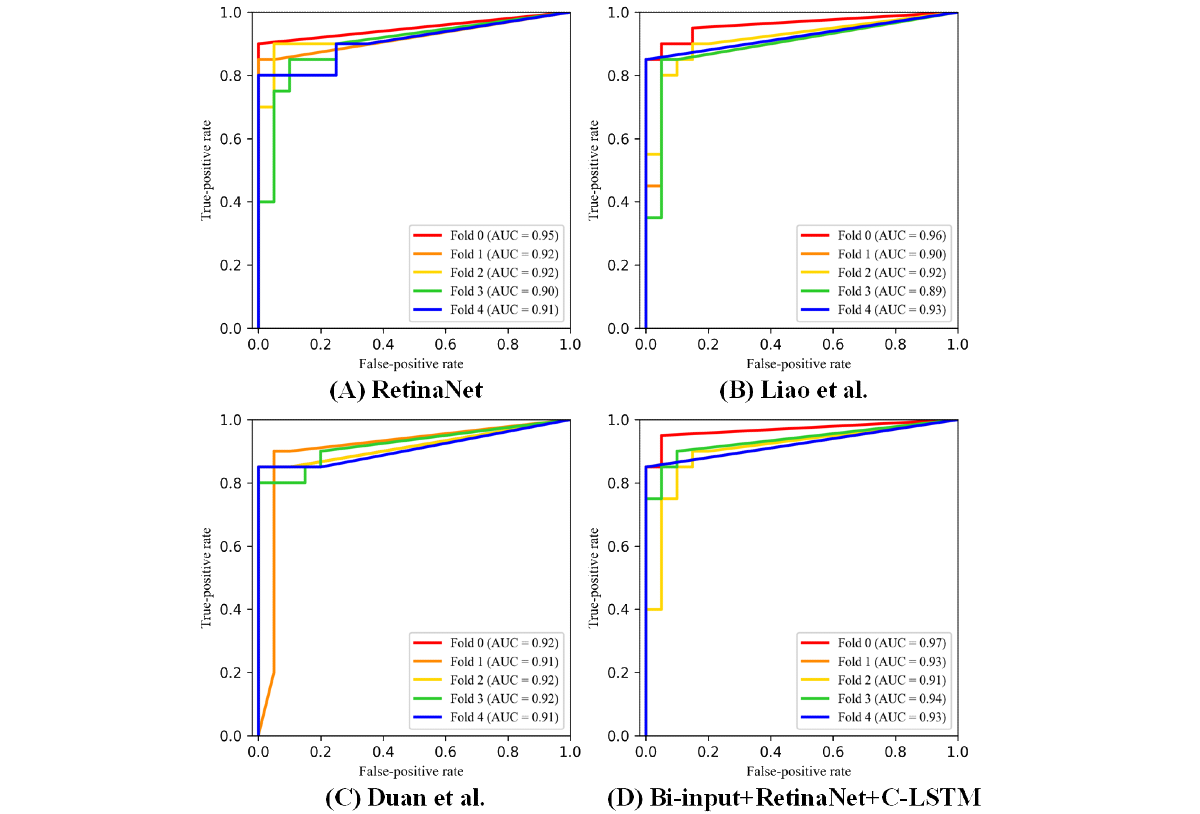
The 5-fold cross-validation results for the ROC curves and AUC values of the different frameworks. The results of different cross-validation models are shown in different colors. A: RetinaNet [25]. B: Liao et al [15]. C: Duan et al [16]. D: Bi-input+RetinaNet+C-LSTM. The ROC curves of fold 0 and fold 2 in graph C overlap, and the ROC curves of fold 1 and fold 4 in graph D overlap. AUC: area under the curve; C-LSTM: convolutional long short-term memory; ROC: receiver operating characteristic.

**Figure 6:**
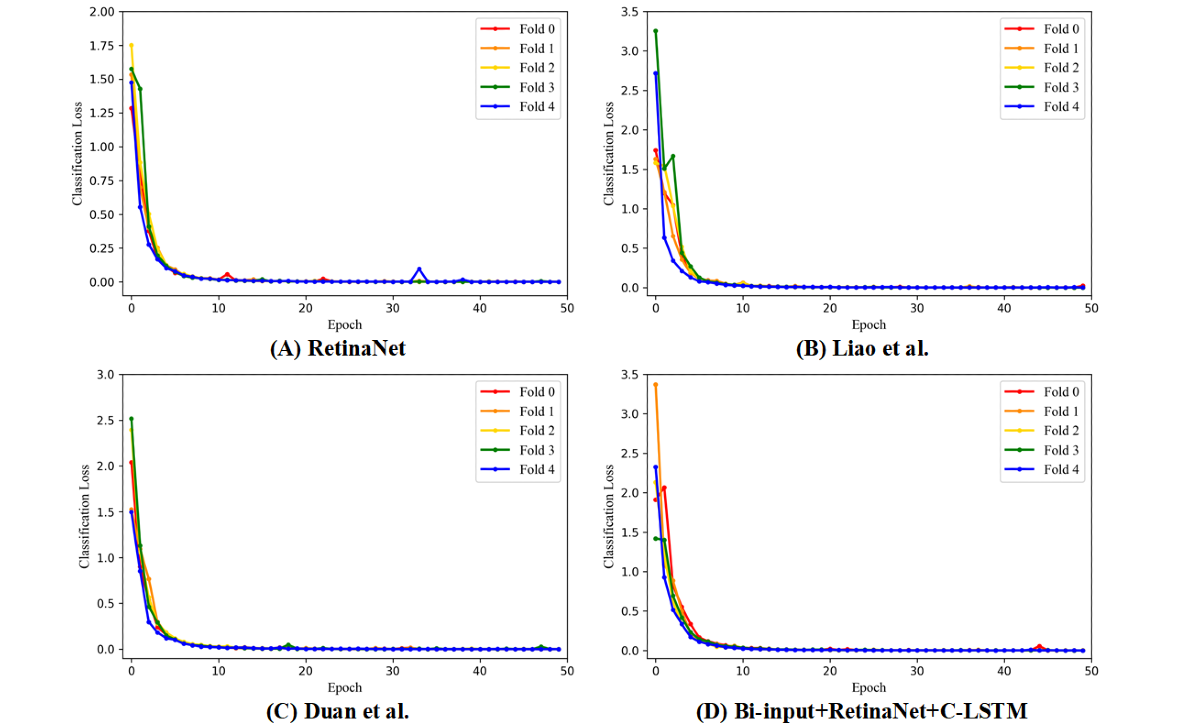
The 5-fold cross-validation results of the focal loss of each framework. Different color curves indicate different cross-validation models. A: RetinaNet [25]. B: Liao et al [15]. C: Duan et al [16]. D: Bi-input+RetinaNet+C-LSTM. C-LSTM: convolutional long short-term memory.

**Figure 7:**
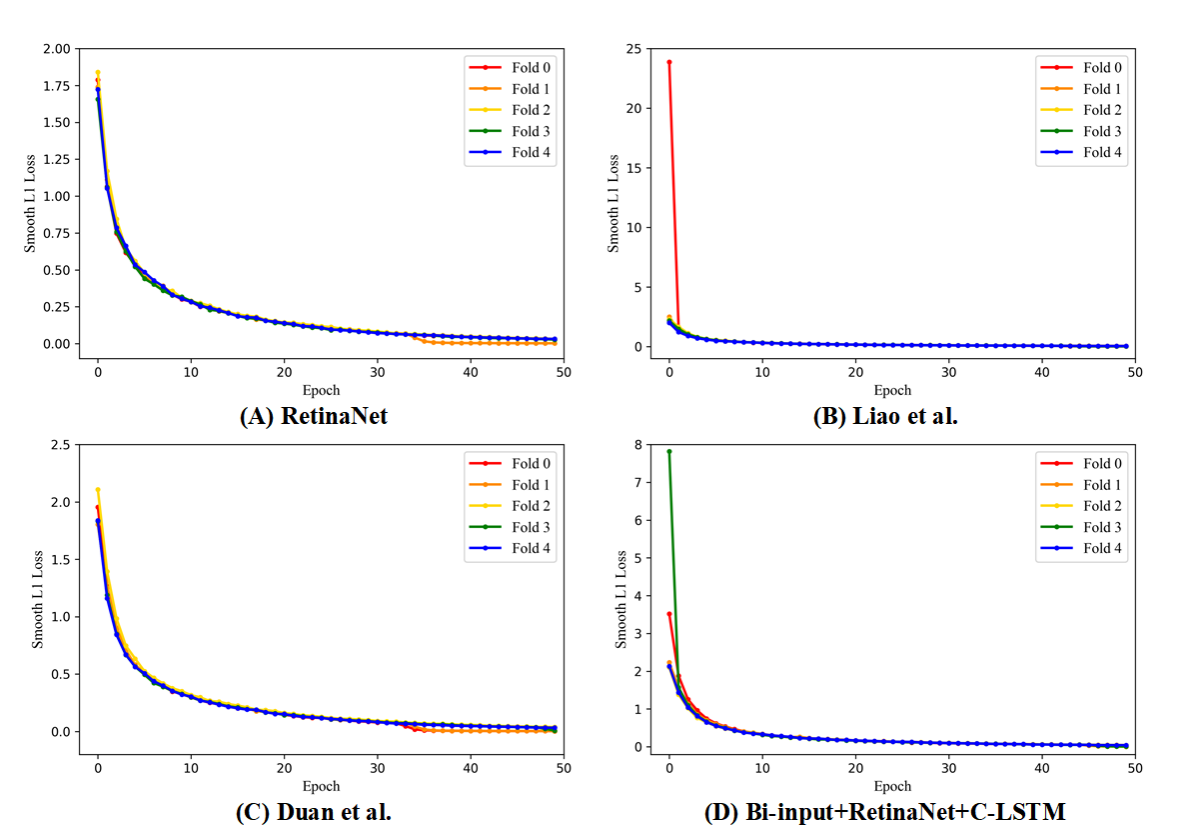
The 5-fold cross-validation results of the smooth L1 loss of each framework. Different color curves indicate different cross-validation models. A: RetinaNet [25]. B: Liao et al [15]. C: Duan et al [16]. D: Bi-input+RetinaNet+C-LSTM. C-LSTM: convolutional long short-term memory.

The AUC values and the ROC curves of RetinaNet [25], Liao et al [15], Duan et al [16], and the bi-input+RetinaNet+C-LSTM framework are shown in Figure 5. The focal loss and the smooth L1 loss also showed that the aforementioned frameworks had sufficient convergence (Figures 6 and 7). Compared to the average AUC values of RetinaNet [25] (0.920), Liao et al [15] (0.920) and Duan et al [16] (0.916), the bi-input+RetinaNet+C-LSTM framework had the largest average AUC value (0.936). The 5-fold cross-validation mAP values of the aforementioned frameworks are listed in Table 1. The mAP represents the average area under the precision-recall curves that were drawn based on the results of aneurysm and blood vessel overlap predictions.

The sensitivity, specificity, and accuracy results of RetinaNet [25], Liao et al [15], Duan et al [16], the bi-input+RetinaNet+C-LSTM framework, and the human experts with 20 years of experience are listed in Table 2.

Compared to the other frameworks’ results, the bi-input+RetinaNet+C-LSTM framework had the best performance. The mean sensitivity, specificity, and accuracy of the bi-input+RetinaNet+C-LSTM framework were 89% (range 67.02%-98.43%), 93% (range 72.30%-99.56%), and 91% (range 77.63%-97.72%), respectively.

**Figure 8:**
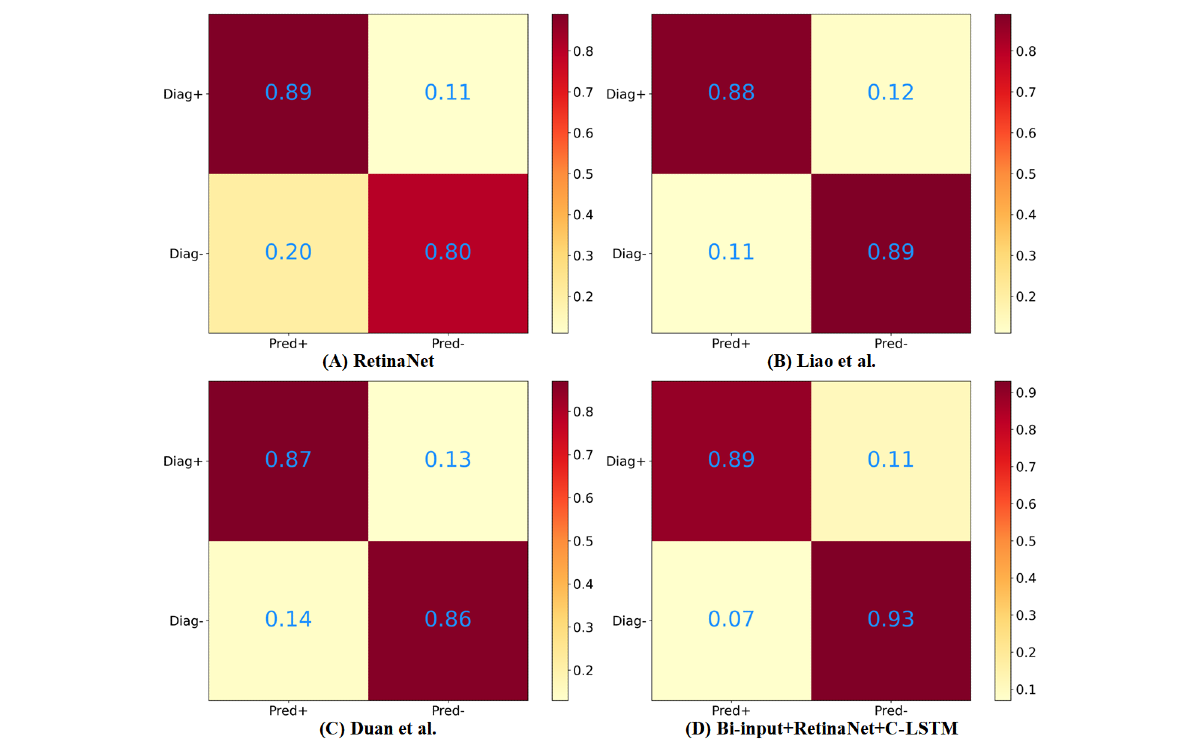
The results of the confusion matrix for each framework. The upper left corners represent true positives, the upper right corners represent false negatives, the lower left corners represent false positives, and the lower right corners represent true negatives. A: RetinaNet [25]. B: Liao et al [15]. C: Duan et al [16]. D: Bi-input+RetinaNet+C-LSTM. C-LSTM: convolutional long short-term memory; Diag+: diagnosed with tumor; Diag-: diagnosed without tumor; Pred+: predicted tumor; Pred-: no predicted tumor.

The confusion matrix of each framework is shown in Figure 8; both the bi-input+RetinaNet+C-LSTM and RetinaNet frameworks had the highest true-positive rate, but the false-positive rate of the bi-input+RetinaNet+C-LSTM framework was much smaller than that of the other frameworks. Therefore, the bi-input+RetinaNet+C-LSTM framework had the best performance compared to that of the other frameworks.

**Figure 9:**
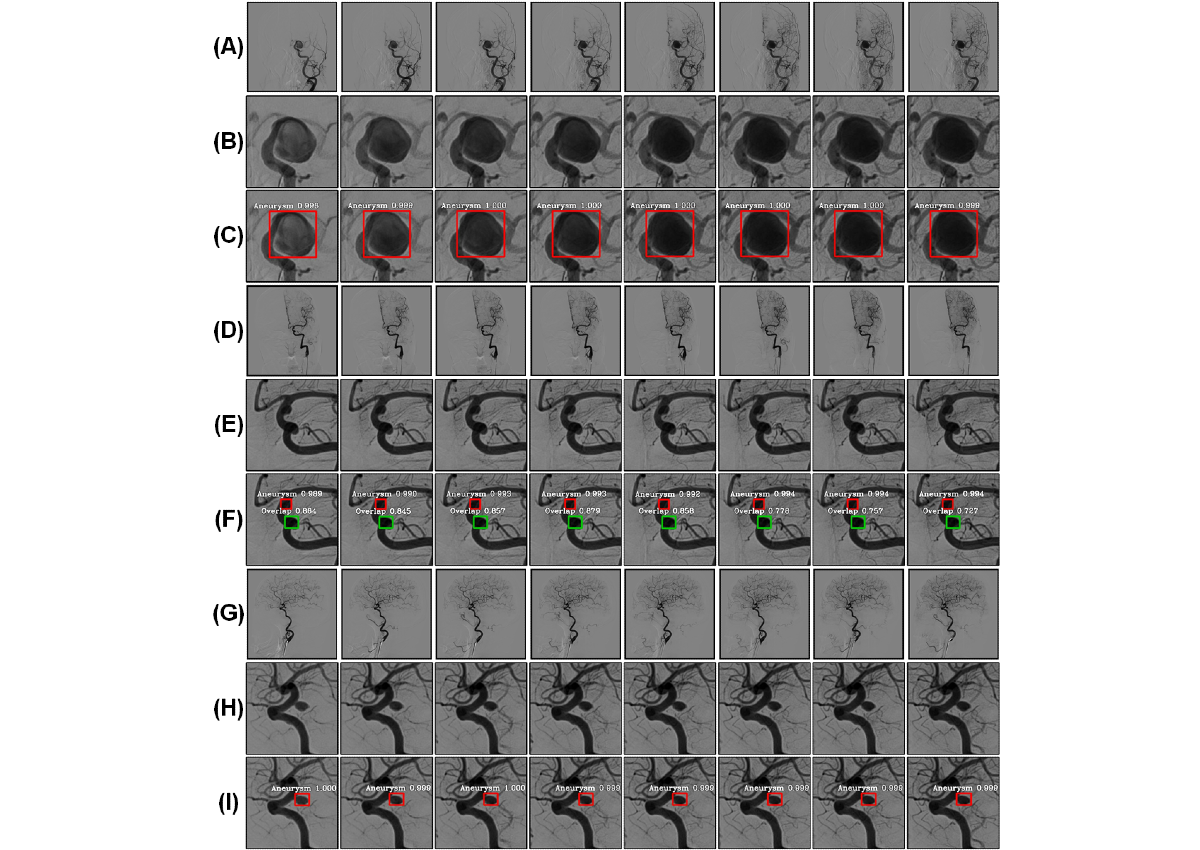
A sample of the original images of the DSA sequence and their corresponding results in the RLS and IADS. A, D, and G represent the raw DSA sequences, and B, E, and H represent the experimental results of the RLS. The results of the IADS are shown in C, F, and I. The red bounding boxes denote the aneurysms, and the green bounding boxes represent the overlapping blood vessels. DSA: digital subtraction angiography; IADS: intracranial aneurysm detection stage; RLS: region localization stage.

The original images of the DSA sequence and their corresponding results for the RLS and IADS are presented in Figure 9, which shows the detection results for different sizes of aneurysms. Most of the results had a confidence level of up to 1.0. This proves that our proposed method performs well in the detection of multiscale aneurysms.

**Table 1:** The mean average precision (mAP) values from the 5-fold cross-validation.

Frameworks	Fold 1, mAP	Fold 2, mAP	Fold 3, mAP	Fold 4, mAP	Fold 5, mAP
RetinaNet [25]	0.4006	0.6553	0.5687	0.6941	0.7569
Liao et al [15]	0.5082	0.6968	0.5852	0.6479	0.7681
Duan et al [16]	0.4982	0.7157	0.4666	0.7925	0.8294
Bi-input+RetinaNet+C-LSTM^a^	0.4435	0.6523	0.5254	0.6506	0.7408

^a^C-LSTM: convolutional long short-term memory.

**Table 2:** The performance of each framework.

Frameworks	Sensitivity (%), mean (range)	Specificity (%), mean (range)	Accuracy (%), mean (range)	Time cost (s)
RetinaNet [25]	89 (67.02-98.43)	80 (56.34-94.27)	84.50 (69.57-93.97)	0.24
Liao et al [15]	88 (65.76-98.06)	89 (67.02-98.43)	88.50 (74.44-96.39)	2.21
Duan et al [16]	87 (64.53-97.66)	86 (63.31-97.24)	86.50 (71.97-95.22)	0.33
Bi-input+RetinaNet+C-LSTM^a^	89 (67.02-98.43)	93 (72.30-99.56)	91 (77.63-97.72)	2.72
Human experts	90 (68.30-98.77)	90 (68.30-98.77)	90 (76.34-97.21)	N/A^b^

^a^C-LSTM: convolutional long short-term memory.

^b^N/A: not applicable.

## Discussion

### Principal Findings

We used the RLS to help decrease the computational load and reduce the interference of unrelated tissues, such as bones and small vessels. This step can reduce time consumption and help neural networks focus on the PCoA region. Moreover, as the 2D DSA images may have had different scales, we used the RLS to standardize the images to the same scale. In the clinical diagnosis process, experienced neurosurgeons and neurointerventional radiologists observed the whole DSA sequence and distinguished overlapping arteries from aneurysms based on the flow of contrast agents through blood vessels. Inspired by this process, we introduced temporal information processing, which has been widely used in text understanding, to improve our diagnostic system. As classic time-processing neural networks, such as LSTM networks, only focus on 1D information, they inevitably result in information loss (ie, the loss of spatial details) when a 2D image is flattened to 1D information. To address this problem, we chose the C-LSTM network, which is specifically designed for 3D data. C-LSTM networks use 3D data as input to process 2D image sequences combined with temporal information. Monodirectional processing methods only allow later features to obtain information from previously inputted images, which results in the imbalance of information. As such, it is difficult to specify which frame might be more important for detection. Bidirectional temporal information processing allows each frame in DSA sequences to combine both past and future information, and each frame can apply the same weight in the diagnosis process. Although processing time information increases detection times, accuracy is more important than speed when it comes to medical imaging tasks. Even if the detection time increases, the model can still complete the detection within 3 seconds, which is acceptable. Therefore, it was reasonable for us to add a bidirectional C-LSTM network to process information.

In the real diagnosis process, physicians often combine the frontal and lateral sequences to make decisions because some aneurysms are difficult to identify in images taken from 1 angle. Based on this idea, we combined the frontal sequences with the lateral sequences together (bi-input) to increase the amount of spatial information and further improve the performance of the diagnostic system. According to the results of this study, the bi-input+RetinaNet+C-LSTM framework improved the sensitivity to 89% and the specificity to 93%, and its accuracy was the highest (91%) among all models. In addition, the bi-input+RetinaNet+C-LSTM framework also had the highest average AUC value and the best confusion matrix. Hence, the bi-input+RetinaNet+C-LSTM framework had the best performance among all models, and its results were similar to those of experienced human experts.

We labeled some overlapping blood vessels that were easily confused with aneurysms, which also indirectly reduced the rate of false positives to some extent. However, adding the overlap labels also caused fluctuations in the mAP values. The reason for this may have been that the physicians only labeled aneurysms and some overlaps, such as the segment of the ICA near the clinoid process. It was difficult to label all of the overlaps, since our main task was to look for aneurysms, and labeling overlaps requires considerable amounts of work. In our framework’s predictions, some overlapping blood vessels were identified by the framework but may not have been marked, and some overlaps were annotated but not detected, which resulted in a large fluctuation in mAP values.

### Conclusion

According to our results, more spatial and temporal information can help improve the performance of the frameworks. Therefore, the bi-input+RetinaNet+C-LSTM had the best performance when compared to that of the other frameworks. Our study demonstrated that our system can assist physicians in detecting intracranial aneurysms on 2D DSA images.

Our experiment had some limitations. First, our data set is comparatively small and only includes PCoA aneurysms. In the future, we will include cerebral aneurysms in different locations. Second, the cascading network framework is relatively complex. Therefore, an end-to-end network should be considered. In future work, we will attempt to find a method that compensates for the loss of information in the process of converting 2D information to 1D information and use a transformer [34] to process time information.

## Abbreviations

AUC: area under the curve
C-LSTM: convolutional long short-term memory
CNN: convolutional neural network
DSA: digital subtraction angiography
FPN: feature pyramid network
IADS: intracranial aneurysm detection stage
ICA: internal carotid artery
LSTM: long short-term memory
mAP: mean average precision
PCoA: posterior communicating artery
ResNet: residual deep neural network
RLS: region localization stage
ROC: receiver operating characteristic
SAH: subarachnoid hemorrhage

## References

[1] Wong JHY, Tymianski R, Radovanovic I, Tymianski M (2015). Minimally invasive microsurgery for cerebral aneurysms. Stroke.

[2] Wardlaw JM, White PM (2000). The detection and management of unruptured intracranial aneurysms. Brain.

[3] Keedy A (2020). An overview of intracranial aneurysms. Mcgill J Med.

[4] Esteva A, Kuprel B, Novoa RA, Ko J, Swetter SM, Blau HM, Thrun S (2017). Dermatologist-level classification of skin cancer with deep neural networks. Nature.

[5] Krizhevsky A, Sutskever I, Hinton GE (2012). ImageNet classification with deep convolutional neural networks. https://papers.nips.cc/paper/2012/file/c399862d3b9d6b76c8436e924a68c45b-Paper.pdf.

[6] Hubel DH, Wiesel TN (1968). Receptive fields and functional architecture of monkey striate cortex. J Physiol.

[7] Fukushima K, Miyake S (1982). Neocognitron: A self-organizing neural network model for a mechanism of visual pattern recognition. Competition and Cooperation in Neural Nets.

[8] Lakhani P, Sundaram B (2017). Deep learning at chest radiography: Automated classification of pulmonary tuberculosis by using convolutional neural networks. Radiology.

[9] Yasaka K, Akai H, Abe O, Kiryu S (2018). Deep learning with convolutional neural network for differentiation of liver masses at dynamic contrast-enhanced CT: A preliminary study. Radiology.

[10] Shi X, Chen Z, Wang H, Yeung DY, Wong WK, Woo WC (2015). Convolutional LSTM network: a machine learning approach for precipitation nowcasting.

[11] Novikov AA, Major D, Wimmer M, Lenis D, Buhler K (2019). Deep sequential segmentation of organs in volumetric medical scans. IEEE Trans Med Imaging.

[12] Liu X, Liu T, Zhang Z, Kuo PC, Xu H, Yang Z, Lan K, Li P, Ouyang Z, Ng YL, Yan W, Li D (2021). TOP-Net prediction model using bidirectional long short-term memory and medical-grade wearable multisensor system for tachycardia onset: Algorithm development study. JMIR Med Inform.

[13] Podgoršak AR, Bhurwani MM, Rava RA, Chandra AR, Ionita CN (2019). Use of a convolutional neural network for aneurysm identification in digital subtraction angiography.

[14] Jin H, Yin Y, Hu M, Yang G, Qin L (2019). Fully automated unruptured intracranial aneurysm detection and segmentation from digital subtraction angiography series using an end-to-end spatiotemporal deep neural network.

[15] Liao J, Duan H, Dai H, Huang Y, Liu L, Chen L, Zhou L (2019). Automatic detection of intracranial aneurysm from digital subtraction angiography with cascade networks.

[16] Duan H, Huang Y, Liu L, Dai H, Chen L, Zhou L (2019). Automatic detection on intracranial aneurysm from digital subtraction angiography with cascade convolutional neural networks. Biomed Eng Online.

[17] Ojemann RG, Crowell RM (1984). Surgical management of cerebrovascular disease. Ann Surg.

[18] Lin TY, Dollár P, Girshick R, He K, Hariharan B, Belongie S (2017). Feature pyramid networks for object detection.

[19] He K, Zhang X, Ren S, Sun J (2016). Deep residual learning for image recognition.

[20] Ren S, He K, Girshick R, Sun J (2017). Faster R-CNN: Towards real-time object detection with region proposal networks. IEEE Trans Pattern Anal Mach Intell.

[21] Zhang K, Liu X, Liu F, He L, Zhang L, Yang Y, Li W, Wang S, Liu L, Liu Z, Wu X, Lin H (2018). An interpretable and expandable deep learning diagnostic system for multiple ocular diseases: Qualitative study. J Med Internet Res.

[22] Ko H, Chung H, Kang WS, Kim KW, Shin Y, Kang SJ, Lee JH, Kim YJ, Kim NY, Jung H, Lee J (2020). COVID-19 pneumonia diagnosis using a simple 2D deep learning framework with a single chest CT image: Model development and validation. J Med Internet Res.

[23] Liang B, Yang N, He G, Huang P, Yang Y (2020). Identification of the facial features of patients with cancer: A deep learning–based pilot study. J Med Internet Res.

[24] Huang P, Yu G, Lu H, Liu D, Xing L, Yin Y, Kovalchuk N, Xing L, Li D (2019). Attention-aware fully convolutional neural network with convolutional long short-term memory network for ultrasound-based motion tracking. Med Phys.

[25] Lin TY, Goyal P, Girshick R, He K, Dollár P (2017). Focal loss for dense object detection.

[26] Long J, Shelhamer E, Darrell T (2015). Fully convolutional networks for semantic segmentation.

[27] Jung H, Kim B, Lee I, Yoo M, Lee J, Ham S, Woo O, Kang J (2018). Detection of masses in mammograms using a one-stage object detector based on a deep convolutional neural network. PLoS One.

[28] Umer J, Irtaza A, Nida N (2021). MACCAI LiTS17 liver tumor segmentation using RetinaNet.

[29] Gräbel P, Özkan Ö, Crysandt M, Herwartz R, Baumann M, Klinkhammer BM, Boor P, Brümmendorf TH, Merhof D (2020). Circular anchors for the detection of hematopoietic cells using Retinanet.

[30] Keras: The Python Deep Learning library. The SAO/NASA Astrophysics Data System.

[31] Kingma D, Ba J Adam: A method for stochastic optimization. arXiv. Preprint posted online on December 22, 2014.

[32] DeLong ER, DeLong DM, Clarke-Pearson DL (1988). Comparing the areas under two or more correlated receiver operating characteristic curves: A nonparametric approach. Biometrics.

[33] Agresti A, Coull BA (1998). Approximate is better than “Exact” for interval estimation of binomial proportions. Am Stat.

[34] Vaswani A, Shazeer N, Parmar N, Uszkoreit J, Jones L, Gomez AN, Kaiser L, Polosukhin I Attention is all you need. arXiv. Preprint posted online on December 6, 2017.

